# Encapsulation of CoS*_x_* Nanocrystals into N/S Co‐Doped Honeycomb‐Like 3D Porous Carbon for High‐Performance Lithium Storage

**DOI:** 10.1002/advs.201800829

**Published:** 2018-07-20

**Authors:** Bo Yin, Xinxin Cao, Anqiang Pan, Zhigao Luo, Selvakumaran Dinesh, Jiande Lin, Yan Tang, Shuquan Liang, Guozhong Cao

**Affiliations:** ^1^ School of Material Science and Engineering Central South University Changsha 410083 China; ^2^ Department of Materials Science & Engineering University of Washington Seattle WA 98195 USA

**Keywords:** anodes, CoS*_x_* nanocrystals, honeycomb‐like porous structures, lithium storage, N/S co‐doped carbon

## Abstract

A honeycomb‐like 3D N/S co‐doped porous carbon‐coated cobalt sulfide (CoS, Co_9_S_8_, and Co_1–_ *_x_*S) composite (CS@PC) is successfully prepared using polyacrylonitrile (PAN) as the nitrogen‐containing carbon source through a facile solvothermal method and subsequent in situ conversion. As an anode for lithium‐ion batteries (LIBs), the CS@PC composite exhibits excellent electrochemical performance, including high reversible capacity, good rate capability, and cyclic stability. The composite electrode delivers specific capacities of 781.2 and 466.0 mAh g^−1^ at 0.1 and 5 A g^−1^, respectively. When cycled at a current density of 1 A g^−1^, it displays a high reversible capacity of 717.0 mAh g^−1^ after 500 cycles. The ability to provide this level of performance is attributed to the unique 3D multi‐level porous architecture with large electrode–electrolyte contact area, bicontinuous electron/ion transport pathways, and attractive structure stability. Such micro‐/nanoscale design and engineering strategies may also be used to explore other nanocomposites to boost their energy storage performance.

## 1 Introduction

The ever‐increasing energy demand and environmental deterioration exert huge pressure on the global energy infrastructure.^1^, ^2^ Renewable‐energy technologies, such as lithium‐ion batteries (LIBs), fuel cells, sodium‐ion batteries (SIBs), and solar cells, have made a significant progress. LIBs have been widely applied in the area of consumer electronics, automotive market, and grid for their high energy/power density and durable cycle life.^3^ However, the use of graphite as conventional anode material is difficult to meet the requirements for the next‐generation LIBs because of its limited theoretical capacity. It is highly desirable to develop new anode candidates with high energy density, long cycling life, as well as environmental friendliness.

Recently, transition‐metal chalcogenides (TMCs) have attracted tremendous attention for its multitude of possible valence states, stoichiometric compositions, and crystal structure.^4^, ^5^, ^6^, ^7^ Compared with their oxide counterparts, TMCs usually exhibit better electrical conductivity, and thermal and mechanical stability.^7^ However, TMC materials undergo serious volume changes during the cycling process, which results in poor cycle stability.^8^ Thus, many strategies have been adopted to improve the performances of electrode materials such as carbon modification,^9^, ^10^, ^11^, ^12^, ^13^ tuning particle morphology,^14^, ^15^, ^16^ electrolyte optimization,^17^ hybridization with other composites,^18^, ^19^ cut‐off voltage control,^20^ and nanonization.^9^, ^10^, ^11^, ^12^, ^13^, ^14^, ^15^, ^16^, ^17^, ^18^, ^19^, ^20^ Among numerous TMCs materials, cobalt sulfide materials with different stoichiometric compositions such as Co_9_S_8_, CoS, Co_3_S_4_, and CoS_2_ are considered as ideal candidates for next‐generation LIB's high‐capacity anodes.^21^

Polyacrylonitrile (PAN) with different kinds of molecular weights is often used in electrospinning to shape it into 1D nanofibers.^22^ In addition, PAN nanofibers are often used as substrates to load other active materials^23^, ^24^ or templates for producing hollow tubular structures.^22^, ^25^ Besides, PAN has many carbon—nitrile (C—CN) bonds that enable in situ nitrogen doping during a high‐temperature pyrolysis and carbonization process.^22^, ^26^ However, PAN is rarely used as raw material for hydrothermal reaction or solvothermal reaction, owing to its solubility in polar organic solvents such as dimethylformamide, dimethyl sulfoxide, sulfolane, and ethylene nitrate but insolubility in water and alcohol.

**Figure 1:**
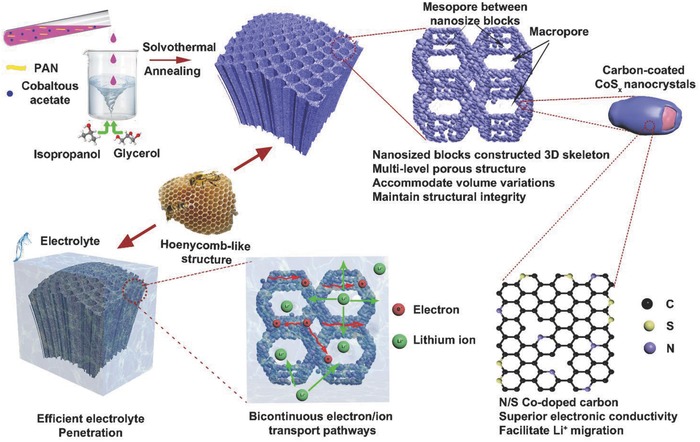
Schematic illustration of honeycomb‐like nitrogen/sulfur co‐doped 3D carbon‐coated porous cobalt sulfide.

Herein, we report the synthesis of honeycomb‐like 3D N/S co‐doped porous carbon‐coated cobalt sulfide (CS@PC) via solvothermal and annealing treatment using PAN as both the carbon source and the nitrogen source (functioned by the carbon—nitrile bonds). The large macroporous structure are constructed of interconnected sheet‐like materials (just like the wall of a honeycomb), and the sheets are built of primary nanosized blocks, between which the mesopores are distributed (**Figure** **1**). This unique 3D multi‐level porous structure can not only ensure sufficient infiltration of the electrolyte, but also can accommodate the volume variations during the discharge/charge process and maintain the structure integrity. In addition, the cobalt sulfide nanocrystals are embedded in the N/S Co‐doped conductive carbon matrix, which endows the superior electronic conductivity. Benefiting from these advantages, the CS@PC composite electrode manifests high reversible capacity, good rate capability, and cyclic stability, making it a promising anode for high‐performance LIBs.

## 2 Results and Discussion

The synthesis of CoS*_x_* nanocrystals embedded into honeycomb‐like N/S co‐doped porous carbon is simple and effective through solvothermal reaction and subsequent in situ conversion. First, PAN and Co(CH_3_COO)_2_·4H_2_O were dissolved in dimethyl formamide (DMF) to form a transparent pink solution with 50 °C water bath. And a certain proportion of glycerol was mixed with isopropyl alcohol to control the viscosity and polarity of the solution, thereby regulating the rate of separating PAN in the mixed solution. Then, DMF mixed solution was added dropwise to the mixed alcohol solution. The moment when the mixed DMF liquid droplets entered the alcohol solution, the surface layer was separated out and precipitated due to the different solubility. The new surface of the droplet also underwent the same process, resulting in the formation of a variety of sheet‐like materials. The Co^2+^ and PAN macromolecules were evenly distributed at the molecular level in solution A, and the entire transformation process was very short‐lived, so Co^2+^ were also uniformly distributed in the PAN precipitation. Due to the isotropy of the precipitation, the surface of the sheet‐like materials forms a 3D multi‐level porous structure at the same time. During the subsequent solvothermal reaction and annealing process, the pore structure was further ripened to eventually form 3D N/S Co‐doped porous carbon‐coated cobalt sulfide.

**Figure 2:**
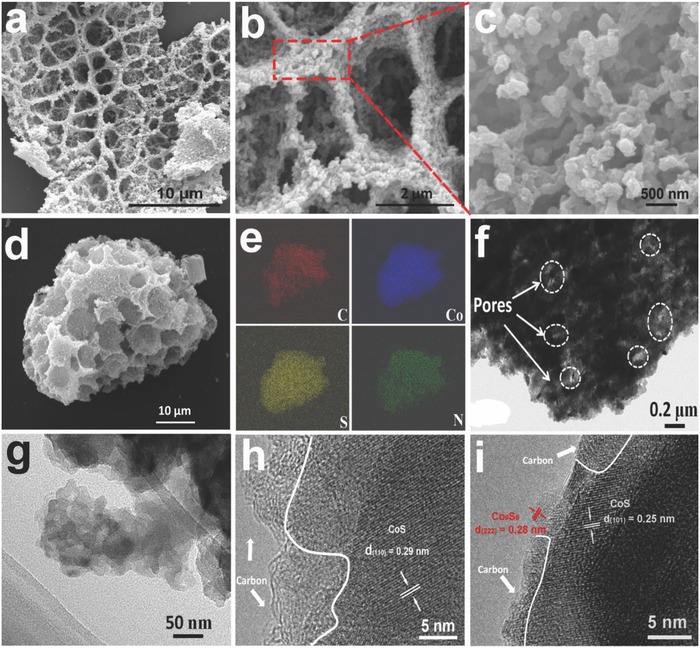
a,b,c) SEM images, d,e) element mapping images (C, Co, S, N), f,g) TEM images, and h,i) the high‐magnification TEM images of CS@PC composite.

In order to study the changes of the pore structure and the secondary nanoparticles in the preparation process, we also observed the precipitated products (without solvothermal reaction, denoted as Co@PAN‐A) and solvothermal products (denoted as Co@PAN‐B). The morphologies and microstructures of Co@PAN‐A, Co@PAN‐B, and CS@PC were investigated by scanning electron microscopy (SEM). From the SEM images, both Co@PAN‐A (Figure S1a,b, Supporting Information) and Co@PAN‐B (Figure S1c,d, Supporting Information) have multi‐level porous structure, and CS@PC (**Figure** **2**a,b) inherits this structure very well. A low‐magnification SEM image (Figure S2, Supporting Information) reveals that the multi‐level pores are homogenously distributed in a large scale. There is no damage of the porous structure during the high‐temperature vulcanization. And the large macroporous structure are constructed of interconnected sheet‐like materials, which have a diameter range of 1–3 µm (Figure 2a). A comparison of Figure S1b (Supporting Information) and Figure S1d (Supporting Information) reveals that the sheets are built of primary nanosized blocks, but Co@PAN‐B sample has larger particles and gap size. The difference in particles size may be attributed to a large number of Co^2+^ that interact with solvents thereby inducing growth and the change of gap size. Figure 2b,c further illustrates that the honeycomb‐like 3D porous structure is composed of interconnected primary nanosized particles which have lager size but smaller gap compared with Co@PAN‐B. These changes may arise from the formation of cobalt sulfide and the pyrolysis and aggregation of PAN macromolecules during high‐temperature annealing. This argument can be demonstrated from the changes in specific surface area and pore size distribution of three samples, which will be discussed later. The elemental composition of the CS@PC is analyzed by EDX analysis as shown in Figure 2d,e. The results give evidence of the coexistence and homogeneous distribution of C, S, Co, and N elements, confirming that cobalt sulfide is uniformly coated by N/S co‐doped carbon.

The pure carbon (denoted as PC) sample (Figure S3, Supporting Information) also has the same 3D porous structure as the CS@PC composite except that it has a smooth sheet structure. This shows that PAN‐coated Co^2+^ contribute to the formation of more complex porous structures by interacting with solvent molecules. The cobalt sulfide without carbon‐coated (denoted as CS) product (Figure S4, Supporting Information) is composed of spherical nanoparticles that are identical to the nanoparticles which make up the honeycomb‐like 3D porous structure of the CS@PC composite except no carbon on the surface, indicating that PAN macromolecules can wrap and bind these nanoparticles together.

Transmission electron microscopy (TEM) and high‐resolution TEM (HRTEM) were used to characterize the microstructure. Figure 2f shows the gaps between nanoparticles, where the light‐colored areas represent holes of different shapes and sizes. Figure 2g further confirms that 3D skeleton is composed of 10–40 nm nanoparticles. The HRTEM image (Figure 2h) shows clear lattice fringes with d‐spacing of 0.29 nm, which correspond to the interplanar of the (1 1 0) plane of hexagonal CoS crystal, and a certain thickness of carbon layer is also observed. By comparing interplanar distances measured from HRTEM with theoretical value, the coexistence of CoS and Co_9_S_8_ is confirmed (Figure 2i).

**Figure 3:**
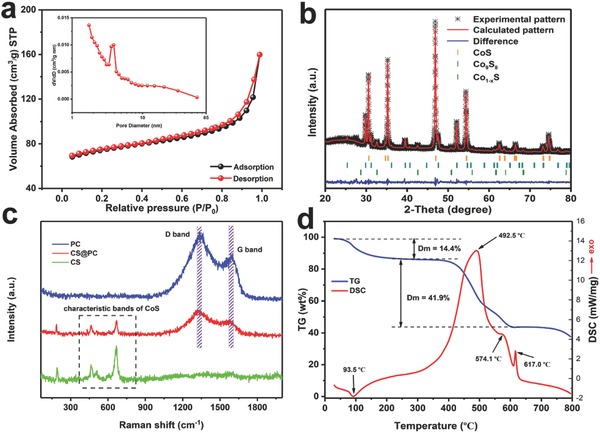
a) N_2_ adsorption–desorption isotherm and b) X‐ray diffraction pattern with Rietveld refinement of CS@PC. c) Raman scattering spectra of PC, CS, and CS@PC and d) TG‐DSC curve of CS@PC.

The nitrogen adsorption–desorption test was performed to investigate the Brunauer–Emmett–Teller (BET) surface area and pore structure of the samples. BET test shows Co@PAN‐A (Figure S5a, Supporting Information) has a surface area of 49.2 m^2^ g^−1^ and pore volume of 0.17 cm^3^ g^−1^ with pore sizes ranging below 10 nm. Co@PAN‐B (Figure S5b, Supporting Information) sample has a lager surface area (174.7 m^2^ g^−1^) and bigger pore volume (0.26 cm^3^ g^−1^) with more concentrated pore distribution. This is consistent with the trend of structural changes analyzed above. With the smooth sheet structure and lower pore volume, PC sample has the smallest BET surface area (Figure S5c, Supporting Information, 13.4 m^2^ g^−1^). With a specific surface area of 73.8 m^2^ g^−1^ for CS sample (Figure S5e, Supporting Information), the composite of CoS*_x_* and PC may have a higher porosity. CS@PC sample (**Figure** **3**a) has the highest specific surface area (234.3 m^2^ g^−1^) and lowest pore volume (0.16 cm^3^ g^−1^), and because PAN macromolecules and organics undergo intense pyrolysis and carbonization under high‐temperature calcination, the pore volume is reduced but the number of mesopores is increased, resulting in an increased surface area. The micropore distribution of CS@PC is also provided in Figure S5f (Supporting Information). Therefore, multi‐level porous structure is constructed by interweaving micropores, mesopores, and macropores. The high surface area and porous structure are believed to be advantages of providing more active sites for electrochemical reaction and easy path for electrolyte penetration.^27^

The Rietveld refinement of X‐ray diffraction (XRD) pattern of the CS@PC is shown in Figure 3b and the calculated pattern matches well with the experimental raw data (*R*_wp_ = 7.76). All diffraction peaks can be indexed to the hexagonal CoS phase (ICSD Card No. 624857), cubic Co_9_S_8_ phase (ICSD Card No. 660368), and trigonal Co_1−_ *_x_*S phase (ICSD Card No. 42703). The mass fraction of CoS, Co_9_S_8_, and Co_1−_ *_x_*S is 75.8, 20.2, and 4.0 wt%, respectively, and Table S1 (Supporting Information) displays the refined cell parameters of the three phases in the prepared CoS*_x_* composite. According to the literature we reviewed, the mixed phase of cobalt sulfide is easily obtained during the preparation^28^, ^29^, ^30^, ^31^ and can be purified by subsequent annealing in air.^32^ Wu et al. obtained a mixed phase of CoS and Co_9_S_8_ by mixing ZIF‐67 with sulfur powder and annealing in an argon atmosphere.^6^ We find two reasons why carbon‐coated cobalt sulfide materials form a mixture phase through our experiments. First, XRD analysis of the CS product (Figure S6c, Supporting Information) indicates that a CoS, Co_9_S_8_, and Co_1−_ *_x_*S mixture phase is kinetically favored and the relative diffraction peak intensity ratio of the (3 1 1) crystal of the Co_9_S_8_ phase to the (1 0 0) of the CoS phase is higher than that of the CS@PC, which indicates the mixing of PAN may prevent the formation of Co_9_S_8_ phase. Second, when the solution B was added dropwise to solution A, the difference in concentration of Co^2+^ between the two solution causes diffusion of Co^2+^ from high to low, which means from solution B (0.189 mol L^−1^) to solution A (0 mol L^−1^). The diffusion process is also carried out during the solvothermal process, resulting in a small amount of precursors that cannot be coated with PAN and eventually forming a Co_9_S_8_ second phase. This conclusion can be corroborated from the HRTEM image (Figure 2i). A careful observation shows that the Co_9_S_8_ nanocrystal is located on the edges of the bulk crystals and are not covered with carbon, compared with the CoS crystal coated with carbon, again demonstrating that carbon coating can inhibit the formation of Co_9_S_8_. We also performed another set of experiments to further verify this conclusion. A weak diffraction peak of Co_9_S_8_ was obtained when 0.1 g Co(CH_3_COO)_2_·4H_2_O was added to solution B (0.134 mol L^−1^) (denoted as 0.1 CS; Figure S6a, Supporting Information). The XRD pattern of the pure carbon material obtained from PAN pyrolysis is shown in Figure S7 (Supporting Information). The two diffraction peaks are corresponding to the (0 0 2) and (1 0 0) crystal planes of carbon. According to the 2θ degree of (0 0 2), the average value of interlayer distance (*d*_002_) is 0.352 nm, which is larger than 0.335 nm of graphite. The expanded interlayer spacing of the carbon material might facilitate the migration of Li^+^.^33^, ^34^

Raman spectroscopy was used to confirm the existence of cobalt sulfide and/or carbon in the final products. The Raman spectra of CS@PC, CS, and PC are presented in Figure 3c. On the one hand, it can be observed that both CS@PC and CS exhibit Raman peaks at 463, 508, and 667 cm^−1^, which are assigned to *E*_g_, *F*_2g_, and *A*_1g_ modes of CoS, respectively.^35^, ^36^ In addition, the peak below 300 cm^−1^ can be indexed to Co_9_S_8_.^37^ On the other hand, CS@PC also presents two characteristic peaks around 1327 and 1574 cm^−1^, which correspond to the D band and G band, respectively, and consistent with PC sample. All these signs indicate that the CS@PC sample is a composite of cobalt sulfide and carbon materials. It is well known that the so‐called D band is associated with amorphous carbon, where defects or edges can break the symmetry and selection rule,^37^ and G band is assigned to the *E*_2g_ vibrations of graphite.^38^, ^39^ The strong D band indicates that lots of disordered sites, heteroatom doping, or defects were existing in the carbon structure of PC and CS@PC.^40^ The peak intensity of *I*_D_/*I*_G_ generally provides a useful index about the degree of crystallinity of carbon materials, that is, the bigger the *I*_D_/*I*_G_ ratio, the higher the degree of disordering in the carbon material.^41^ The *I*_D_/*I*_G_ values of PC and CS@PC are 1.29 and 1.22, respectively, indicating the carbon in both samples have low crystallinity, which is caused by nitrogen and sulfur co‐doping.^40^ The incorporation of nitrogen and sulfur into carbon can increase the conductivity of the material and thus improve its electrochemical performance.

Thermogravimetric (TG) and differential scanning calorimetry (DSC) analysis was carried out to quantify the content of components in the composites. Three samples were heated from room temperature to 800 °C with a heating rate of 10 °C min^−1^ under air atmosphere (gas flow rate: 25 mL min^−1^), and the results are shown in Figure 3d and Figure S8 (Supporting Information). The PC sample (Figure S8a, Supporting Information) has the simplest TG curve with a complete loss of mass due to the oxidation of carbon in the range of 470–690 °C. Figure 3d and Figure S8c (Supporting Information) show the TG curves of CS@PC and CS, respectively. There are three stages of weight loss for CS@PC composite. The first weight loss appears at approximately 50–150 °C, which can be attributed to the evaporation of physically adsorbed water in the sample.^12^, ^42^ The second loss takes place at about 380–500 °C, signifying the partial combustion of the coated carbon in CS@PC.^30^ The third one arises at approximately 500–610 °C, which is attributed to the used‐up residual carbon and the oxidation of cobalt sulfide to cobalt sulfate.^16^, ^43^ Consequently, the quality loss rate is smaller than the second stage. The carbon of CS@PC sample is completely burned between 380 and 610 °C, which is lower than that for the PC sample. This may be due to the fact that the CS@PC sample has smaller grains and higher specific surface area and lager contact reaction area with air, so the surface‐coated carbon is oxidized at a lower temperature. For the TG curve of CS sample, there is a significant increase in quality at both temperature range of 210–420 °C and 450–610 °C, which correspond to the gradual conversion of cobalt sulfide to cobalt sulfate.^16^, ^43^, ^44^ In addition, the weight loss between 420 and 450 °C is due to the consumption of carbon from solvothermal reaction, corresponding to the strong exothermal peak around 476.1 °C. The XRD patterns of the residual materials after TG‐DSC tests of CS@PC (Figure S8b, Supporting Information) and CS (Figure S8d, Supporting Information) were collected. All the diffraction peaks are indexed well to CoSO_4_ phase (JCPDS card: 82–0185) and Co_3_O_4_ phase (JCPDS card: 74–2120), indicating that the little mass loss above 700 °C is due to a slight decomposition of CoSO_4_. By assuming that the remaining product around 700 °C of the TG‐DSC measurement is pure CoSO_4_, with a weight percentage of approximately 43.7%, we can estimate that the cobalt sulfide (calculated based on CoS) content in the CS@PC is about 29.9%.

**Figure 4:**
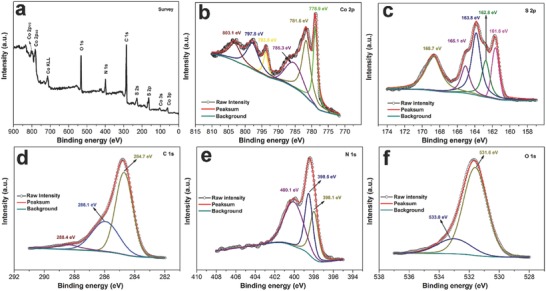
a) Typical XPS survey spectrum and the corresponding b) Co 2p, c) S 2p, d) C 1s, e) N 1s, and f) O 1s spectra of the CS@PC.

X‐ray photoelectron spectroscopy (XPS) measurements were conducted to ascertain the electronic structure and surface chemical compositions of the CS@PC sample. As shown in **Figure** **4**a, the survey scan spectrum suggests that CS@PC composite is mainly composed of cobalt, sulfur, carbon, nitrogen, and oxygen. The high‐resolution spectrum of Co 2p (Figure 4b) displays four main peaks: two peaks centered around binding energies 778.9 and 793.8 eV are attributed to the Co—S bond and two peaks at 781.6 and 797.8 eV belong to the Co—O bond.^16^, ^45^ The oxygen element in the CS@PC composite may arise from three parts: the absorbed hydroxide species, precursor residual oxygen after vulcanization, and sulfur species from partial oxidation of material surface.^40^, ^46^, ^47^ Besides, the two satellite peaks at 785.3 and 803.1 eV could be indexed to the shake‐up peak of Co^2+^.^16^ As for the S 2p core‐level XPS spectrum (Figure 4c), the peaks at 161.6 and 162.8 eV are assigned to Co‐S 2p_3/2_ and 2p_1/2_, respectively, indicating the formation of CoS*_x_*.^47^, ^48^ And the peak at 168.7 eV corresponds to SO*_x_*, reflecting the presence of oxygen in the sample again.^49^ The other two peaks at 163.8 and 165.1 eV could be identified as C‐S 2p_3/2_ and 2p_1/2_, indicating the successful sulfur doping of the carbon. In the case of C (Figure 4d), the peaks centered at 284.7, 286.1, and 288.4 eV can be attributed to C—C, C—S/C—N, and C—N bonds, respectively.^46^, ^48^, ^50^ The presence of C—N bond demonstrates that nitrogen is doped into the carbon lattice. The existence of nitrogen element can be confirmed by N 1s spectra (Figure 4e), including pyridinic‐, pyrrolic‐, and graphitic‐type nitrogen in carbon matrix at binding energies of 398.1, 398.5, and 400.1 eV, respectively, which provide additional evidence of successful doping of nitrogen into carbon.^7^, ^47^ The doped nitrogen atoms can not only improve the electronic conductivity of carbon, especially pyrrolic N and pyridinic N, but also provide active sites for the growth of cobalt sulfur nanoparticles because they can influence the electronic properties of the surrounding carbon substrate and act as electron donors.^7^, ^46^, ^51^ In the O 1s spectrum (Figure 4f), the peak at 533.0 eV is corresponding to the Co—O bond, and the intensive peak at 531.6 eV is owing to a high number of defect sites with a low oxygen coordination, indicating that a large number of oxygen vacancies existed in the CS@PC composite. Oxygen vacancies can enhance material's electric conductivity and improve Li^+^ diffusion coefficient by introducing more defects and distorted lattices.^52^, ^53^ The synergistic effect of N/S co‐doped and oxygen vacancies may be beneficial to boost the electrochemical performance of the CS@PC composite.

For comparison, the high‐resolution spectra of C 1s, N 1s, and S 2p and the survey spectrum of pure carbon annealed with sulfur powder (denoted as PC‐S) are shown in Figure S9 (Supporting information), further conforming the successful sulfur and nitrogen doping of carbon. In addition, the elemental compositions of CS@PC and PC‐S are listed in Table S2 (Supporting Information). As we can see, the number of sulfur atoms is more than twice the cobalt atoms, indicating that sulfur is present not only in CoS*_x_* but also through doping in the surface carbon materials in the CS@PC composite.

**Figure 5:**
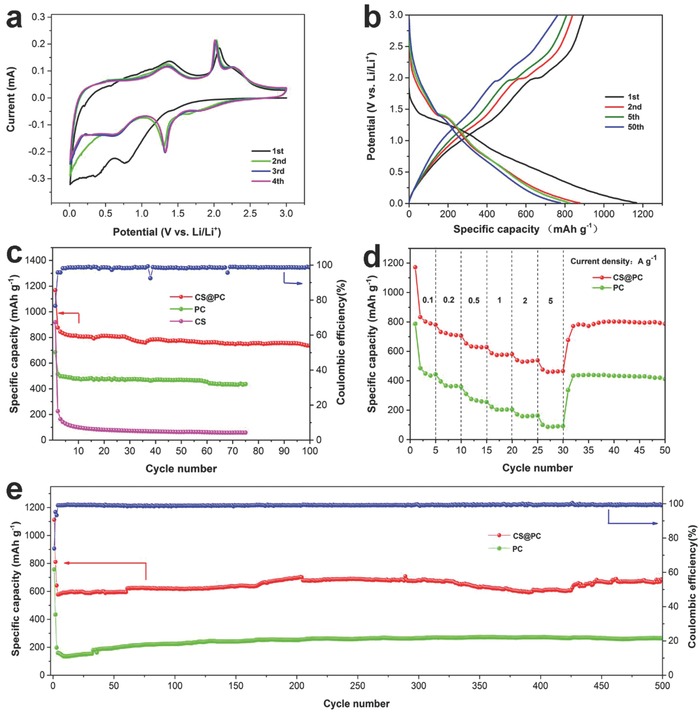
a) The first four successive CV curves of CS@PC and b) the charge–discharge curves of selected cycles for CS@PC at 100 mA g^−1^. c) The cycling performance of the CS@PC, PC, and CS electrodes at 100 mA g^−1^ between 0.01 and 3 V. d) The rate performance of CS@PC and PC at different current densities. e) Long cycle test of the CS@PC and PC electrodes at 1000 mA g^−1^.

The as‐prepared CS@PC, CS, and PC samples were assembled into coin cells to evaluate their electrochemical performance. **Figure** **5**a shows the first four successive cyclic voltammetry (CV) curves of the CS@PC between 0.01–3 V (vs Li^+^/Li) at a scan rate of 0.1 mV s^−1^. For the first cycle in the cathodic process, there is a broad peak at 0.76 V and a small peak near 0.35 V which can be assigned to the conversion of CoS*_x_* with Li and the formation of solid–electrolyte interphase (SEI) film, respectively.^49^ In the next three sweeps, the broad cathodic peak splits into three peaks around 0.63, 1.33, and 1.65 V, corresponding to the multi‐step electrochemical reactions during lithiation. During the anodic process after the first sweep, the three obvious peaks at 1.35, 2.01, and 2.27 V can be ascribed to the reverse reaction to form the CoS*_x_* in the electrode.^46^ Combined with the relevant literature, the total electrochemical reaction could be described as follows:^46^, ^49^

(1) $${\mathrm{CoS}}_{x}\;+\;2x{\mathrm{Li}}^{+}+\;2xe^{-}\;↔\;\mathrm{Co}\;+\;x{\mathrm{Li}}_{2}S$$

The third and fourth cycles are well overlapped, suggesting that the good reversibility of the CS@PC electrode. Figure 5b shows the charge–discharge curves of selected cycles for the CS@PC electrode at 100 mA g^−1^. In the first cycle, the CS@PC composite delivers a discharge capacity of 1167.8 mAh g^−1^ and a reversible charge capacity of 895.5 mAh g^−1^ with a coulombic efficiency of 76.7%. The low initial coulombic efficiency and the large irreversible capacity loss may be ascribed to the formation of SEI on the surface of the electrode materials, irreversible phase transition, and oxygen‐containing functional group of carbon.^21^, ^54^, ^55^ The charge–discharge curves of the fifth and fiftieth cycles are almost overlapping, indicating the improved reversibility of the electrode materials. Figure 5c shows the cycling performance of the CS@PC, PC, and CS samples at 100 mA g^−1^. Both CS@PC and PC display good cycling stability. The reversible capacity of CS@PC is 736.4 mAh g^−1^ after 100 cycles, while PC maintains the capacity of 436.8 mAh g^−1^ after 75 cycles. However, the cycle performance of the CS is extremely poor. The capacity of the tenth circle has been reduced to 99 mAh g^−1^, although its initial capacity is 918.3 mAh g^−1^, indicating the existence of honeycomb‐like 3D porous structure and the N/S co‐doped carbon coating can significantly improve the cycle stability. Figure 5d shows the rate capability of the CS@PC and CS at the current densities of 100, 200, 500, 1000, 2000, and 5000 mA g^−1^. The CS@PC composite delivers specific capacities of 781.2, 706.5, 628.3, 580.3, 538.6, and 466.0 mAh g^−1^, respectively, and the PC sample release the discharge capacities of 394.9, 358.8, 255.3, 205.2, 162.8, and 91.7 mAh g^−1^, respectively. When the current density is reset to 100 mA g^−1^, the capacity can be recovered to 772 and 434.9 mAh g^−1^ for CS@PC and PC, respectively, indicating the excellent rate capability. The specific capacity and rate capability for CS@PC composite are better than most of the state‐of‐art reported cobalt sulfide anodes (Figure S10, Supporting Information). To further confirm the excellent performance of the CS@PC electrode, the long‐term cycling performance of the CS@PC and PC samples was studied by charging/discharging at a current density of 1000 mA g^−1^ (Figure 5e). The first two cycles were tested at 100 mA g^−1^ to fully activate the batteries. CS@PC and PC can deliver specific discharge capacities of 717.0 and 264.1 mAh g^−1^ after 500 cycles, respectively, which is higher than the third cycle (635.9 and 197.5 mAh g^−1^). The increasing capacity may be due to the stepwise activation of the active material to generate more active sites and defects in the honeycomb‐like 3D porous structure for lithium ions storage.^56^ Moreover, the coulombic efficiency of CS@PC electrode is close to 99.5% after the first three cycles, indicating the excellent and stable reversibility. When the loading of the active material is increased to 1.1 mg cm^−2^ (based on the mass of CS@PC), a discharge specific capacity of 679.1 mAh g^−1^ still can be retained after 200 cycles at a current density of 1000 mA g^−1^ (Figure S11, Supporting Information), further demonstrating the good electrochemical performance of the active material. The superior cycling performance can be attributed to the 3D N/S co‐doped porous carbon‐coated cobalt sulfide composite, in which the carbon can improve the stability of electrode materials and enhance the electronic conductivity. Moreover, the porous structure can provide buffer space for the volume changes, and keep the integrity of the structure.^27^ Figure S12 (Supporting Information) shows the SEM images of CS@PC electrode after 500 cycles at 1000 mA g^−1^. The porous structure of CoS*_x_* can still be observed, demonstrating the excellent structural stability of the CS@PC composite as anode for LIBs.

**Figure 6:**
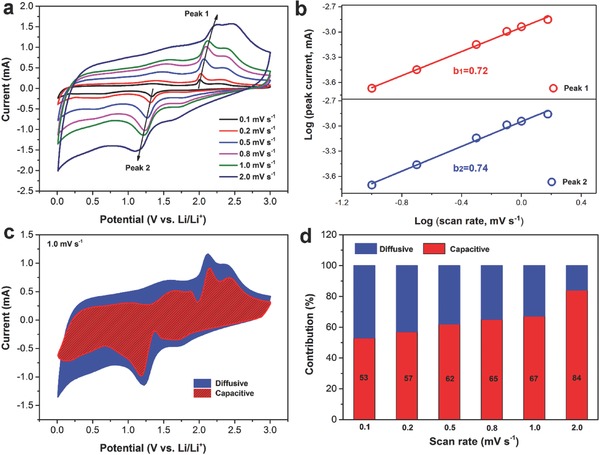
a) CV curves at different scan rates of the CS@PC electrodes and b) corresponding log(*I*) versus log(*v*) plots at specific peak currents. c) Capacitive contribution (red) and diffusion contribution (blue) at 1.0 mV s^−1^. d) Normalized contribution ration of capacities at different scan rates.

Dunn and co‐workers have already proposed that materials with very high surface areas and sophisticated architectures are extrinsic pseudocapacitors.^57^ To investigate the excellent high‐rate performance of CS@PC, the redox pseudocapacitance‐like contribution was analyzed according to the following equations:

(2) $$I\left(V\right)\;=\;av^{b}$$

(3) $$I\;\left(V\right)=k_{1}\;v+k_{2}v^{1/2}$$

where *v* is the sweep rate (mV s^−1^) of a cyclic voltammogram and *I*(*V*) is the current (mA) at the corresponding *v*.^58^ The CV curves of the electrode at different scan rates from 0.1 to 2.0 are plotted in **Figure** **6**a. Obviously, the shape of the CV curves is well preserved with increased scan rates. Equation (2) displays the power–law relationship between *I* and *v*, where *a* and *b* are constants. The value of *b* varies from 0.5 (diffusion‐controlled process) to 1.0 (capacitive‐controlled process) and is calculated by the slope of log (*I*) versus log (*v*) plot (Figure 6b).^59^ And the *b* values for peaks 1 and 2 are 0.72 and 0.74, respectively, indicating surface capacitive‐controlled process kinetically favored the CS@PC electrode.^60^, ^61^ According to Equation (3), by plotting *I*(*V*)/*v* ^1/2^ versus *v* ^1/2^ at different potentials, one can calculate the values of *k*_1_ (slope) and *k*_2_ (intercept) from the straight lines. We can distinguish the fraction of the current from surface capacitance and Li^+^ semiinfinite linear diffusion.^62^ As a result, 67% of the total capacity is identified as the capacitive contribution at the scan rate of 1 mV s^−1^ (Figure 6c). Figure 6d summarizes the percentage of the capacitive contribution of CS@PC composites at different scan rates. With the increase of the scan rate, the diffusion contribution is depressed, while the capacitive contribution is increased as expected.^60^ The high surface area, good 3D conductive network, and abundant active sites make the CS@PC composite with a rather high capacitive contribution, which means that it can withstand the impact of higher density currents, resulting in the CS@PC composite with a superior rate performance.^63^

**Figure 7:**
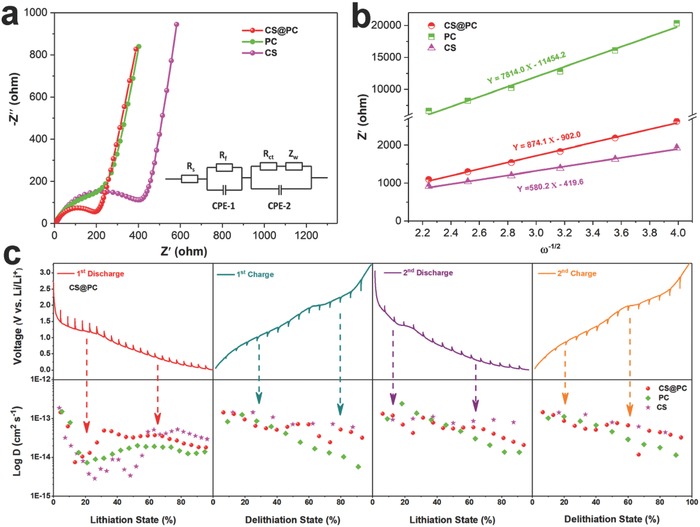
a) EIS spectra of CS@PC, CS, and PC electrodes, and the corresponding equivalent circuit (inset of (a)). b) The relationship plot of *Z*′ versus ω^−1/2^ at low‐frequency region. c) GITT curves and corresponding Li^+^ diffusion coefficient at different discharge/charge states of the CS@PC electrode.

To better understand the excellent electrochemical performance of the CS@PC composite, electrochemical impedance spectroscopy (EIS) and galvanostatic intermittent titration technique (GITT) tests were performed. Nyquist plots (100 kHz–0.01 Hz) of CS@PC, PC, and CS are displayed in **Figure** **7**a. All three samples' plots have similar shapes with a straight line in the low‐frequency region accompanied by a semicircle in the high frequency region. The equivalent circuit model (Figure 7a, inset) is used to better understand the impedance spectrum, which is composed of *R*_s_ (the sum of electrolyte resistance and ohmic resistances of the cell components), *R*_f_ (film resistance), *R*_ct_ (charge‐transfer resistance), CPE‐1 (the constant phase element of the SEI film), CPE‐2 (the double‐layer capacitance), and *Z*_w_ (Warburg impedance).^18^, ^64^, ^65^ The primary fitting parameters of three samples are listed in Table S3 (Supporting Information). Obviously, the composite of cobalt sulfur and porous carbon has a smaller *R*_ct_ value than the pure CoS*_x_*, because N/S co‐doped porous carbon has superior electronic conductivity with the smallest charge‐transfer resistance (PC sample, 106.4 Ω). According to Equation (S1) (Supporting Information), the lithium ions diffusion coefficient is inversely proportional to σ, where σ is the slope of the line *Z*′ − ω^−1/2^.^66^ As shown in Figure 7b, contrary to the EIS pattern, the PC and CS samples have the maximum and minimum slope values, respectively, so the composite of pure cobalt sulfide and porous carbon has a moderate slope, which means a lager lithium ions diffusion coefficient. This outcome is consistent with the GITT test, which was performed to elucidate the effect of multi‐step lithiation/delithiation on ion diffusion and conductivity properties.^61^ The corresponding diffusion coefficients at different discharge/charge voltages were calculated according to Equation (S2) (see details in the Supporting Information), and the results of the first two laps are shown in Figure 7c. It can be clearly seen that CS sample has the highest average diffusion coefficient, but its capacity rapidly decays during cycles (Figure 5c) due to its extremely poor structure stability and electronic conductivity. Although the average diffusion coefficient of the CS@PC sample is slightly smaller than that of CS, it has a porous structure and N/S co‐doped carbon coating, giving its excellent electronic conductivity and structure stability, resulting in a superior rate and a long cycling performance.

## 3 Conclusion

In summary, we have successfully prepared PAN‐coated porous structure cobalt precursor through a facile solvothermal method using PAN as nitrogen‐containing carbon source. The cobalt sulfide nanoparticles coated by the N/S co‐doped carbon are in situ formed by mixing the precursor to react with sulfur powder at 700 °C for 5 h in Ar. The honeycomb‐like 3D porous structure facilitates the electrolyte penetration, buffers the volume changes of active materials, and provides abundant active sites for lithium‐ion storage. Moreover, the N/S co‐doped carbon can not only improve the conductivity of the material, but also work as high‐efficient 3D electron transform pathways. With this unique structure and configuration, the CS@PC electrode exhibits enhanced diffusion kinetics and pseudocapacitive behavior. When evaluated as an anode material for LIBs, the CS@PC electrode displays a high reversible capacity of 717.0 mAh g^−1^ after 500 cycles at a current density of 1000 mA g^−1^. The CS@PC composite also displays excellent rate capability up to 5000 mA g^−1^ for LIBs. Furthermore, this study provides a novel route for the synthesis of 3D porous structure material using PAN as the addition agent, and this smart nanoscale engineering strategy may also be used to explore other advanced materials used in supercapacitors, electrocatalysis, SIBs, and solar cells.

## 4 Experimental Section

*Materials Synthesis*: In a typical procedure, first, a 30 mL mixed solution of glycerol and isopropanol in a volume ratio of 1:10 was prepared and stirred for 0.5 h. The obtained solution was denoted as A. Second, 0.1 g of PAN (*M_w_* = 150 000) and 0.1414 g Co(CH_3_COO)_2_ · 4H_2_O were dissolved in 3 mL *N*,*N*‐dimethyl formamide under stirring for 1 h with 50 °C water bath. The as‐obtained pink solution was denoted as B. Then, the obtained solution B was added drop by drop to solution A under vigorous stirring and after completion of the infusion of solution B, the resultant solution was kept at stirring for another 20 min. The as‐obtained suspension was transferred into a 50 mL Teflon autoclave and kept in an electrical oven at 180 °C for 6 h. After cooling down naturally, the light brown precipitates were collected by centrifugation and washed with absolute alcohol for several times, followed by drying at 80 °C for 24 h. The above prepared precursor was mixed with 0.5 g sulfur powder and annealed under Ar atmosphere at 700 °C for 5 h with a ramping rate of 3 °C min^−1^. The obtained product was denoted as CS@PC. For comparison, the cobalt sulfide without carbon‐coating (denoted as CS) was prepared using the same synthesis strategy, but only 0.1414 g Co(CH_3_COO)_2_ · 4H_2_O was dissolved in 3 mL DMF for solution B. The pure carbon material from PAN pyrolysis (denoted as PC) was prepared by annealing the solvothermal product which had no cobalt source in solution B at 700 °C for 5 h under Ar atmosphere. In addition, sulfur‐doped carbon (denoted as PC‐S) was obtained under the same conditions except 0.5 g sulfur powder was mixed with the solvothermal product before annealing in Ar.

*Materials Characterization*: The crystallographic phase of the product was identified by Powder XRD (Rigaku D/max 2500 X‐ray diffractometer with non‐monochromated Cu Kα radiation, λ = 1.54178 Å). TG and DSC analyses were carried out on a combined DSC and TG analysis instruments (Netzsch STA 449C, Germany). The morphologies of the products were obtained by field emission scanning electron microscopy (FEI Nova NanoSEM 230) and TEM (JEOL‐JEM‐2100F). Nitrogen adsorption–desorption measurements were conducted at 77 K (Micromeritics ASAP 2460). The property of carbon layer was examined by Raman spectrometer (LabRAM HR800). XPS measurements were performed on an ESCALAB 250Xi (ThermoFisher‐VG Scientific, Britain) to probe the oxidation states of elements in the surface.

*Electrode Fabrication and Electrochemical Measurements*: To prepare the working electrode, the active material was mixed with Super P and sodium carboxymethyl cellulose at the weight ratio of 8:1:1 in deionized water to form a slurry, which was then coated on copper foil and dried in a vacuum oven at 80 °C for 12 h. The mass loading of the active material was around 0.8 mg cm^−2^ (based on the weight of CS@PC). The electrodes were assembled into CR2016‐type coin cells in a glovebox (Mbraun, Germany) filled with ultra‐high‐purity argon. Lithium foil was used as the counter and reference electrodes, whereas 1 m LiPF_6_ in ethylene carbonate/dimethyl carbonate/ethyl methyl carbonate (EC/DMC/EMC, 1:1:1, vol%) was used as electrolyte. CV was tested using an electrochemical workstation (CHI 660E) at a scan rate of 0.1 mV s^−1^ in the voltage range of 0.01–3.0 V (vs Li^+^/Li). Galvanostatic charge/discharge behavior was carried out on a multichannel battery testing system (LAND CT2001A, Wuhan, China). The EIS of the electrodes were recorded at room temperature using an electrochemical workstation (MULTI AUTOLAB M204, Metrohm) in the frequency range of 100 kHz–10 mHz on a cell under the as‐assembled condition.

## Conflict of Interest

The authors declare no conflict of interest.

## Supporting information

Supplementary
