# Supplementary material for: Encapsulation of CoSx Nanocrystals into N/S Co‐Doped Honeycomb‐Like 3D Porous Carbon for High‐Performance Lithium Storage
Source: Adv Sci (Weinh). 2018 Jul 20;5(9):1800829. doi: 10.1002/advs.201800829 (PMC6145217; doi:10.1002/advs.201800829)
Supplement: Supplementary file 1 — Supplementary [file ADVS-5-1800829-s001.pdf]

Copyright WILEY-VCH Verlag GmbH & Co. KGaA, 69469 Weinheim, Germany, 2018.

ADVANCED SCIENCE
Open Access

Supporting Information

for *Adv. Sci.*, DOI: 10.1002/advs.201800829

Encapsulation of CoS_x Nanocrystals into N/S Co-Doped Honeycomb-Like 3D Porous Carbon for High-Performance Lithium Storage

Bo Yin, Xinxin Cao, Anqiang Pan,* Zhigao Luo, Selvakumaran Dinesh, Jiande Lin, Yan Tang, Shuquan Liang,* and Guozhong Cao*

Supporting Information for

Encapsulation of CoS_x Nanocrystals into N/S Co-Doped Honeycomb-Like 3D Porous Carbon for High-Performance Lithium Storage

Bo Yin, Xinxin Cao, Anqiang Pan, Zhigao Luo, Selvakumaran Dinesh, Jiande Lin, Yan Tang, Shuquan Liang*, Guozhong Cao**

B. Yin, Dr. X. X. Cao, Prof. A. Q. Pan, Dr. Z. G. Luo, Dr. S. Dinesh, J. D. Lin, Prof. Y. Tang, Prof. S. Q. Liang, Prof. G. Z. Cao

School of Material Science and Engineering

Central South University

Changsha 410083, China

Prof. G. Z. Cao

Department of Materials Science & Engineering

University of Washington

Seattle 98195, USA

E-mail: pananqiang@csu.edu.cn (A. Pan); lsq@csu.edu.cn (S.Q. Liang); gzcao@u.washington.edu (G. Z. Cao)

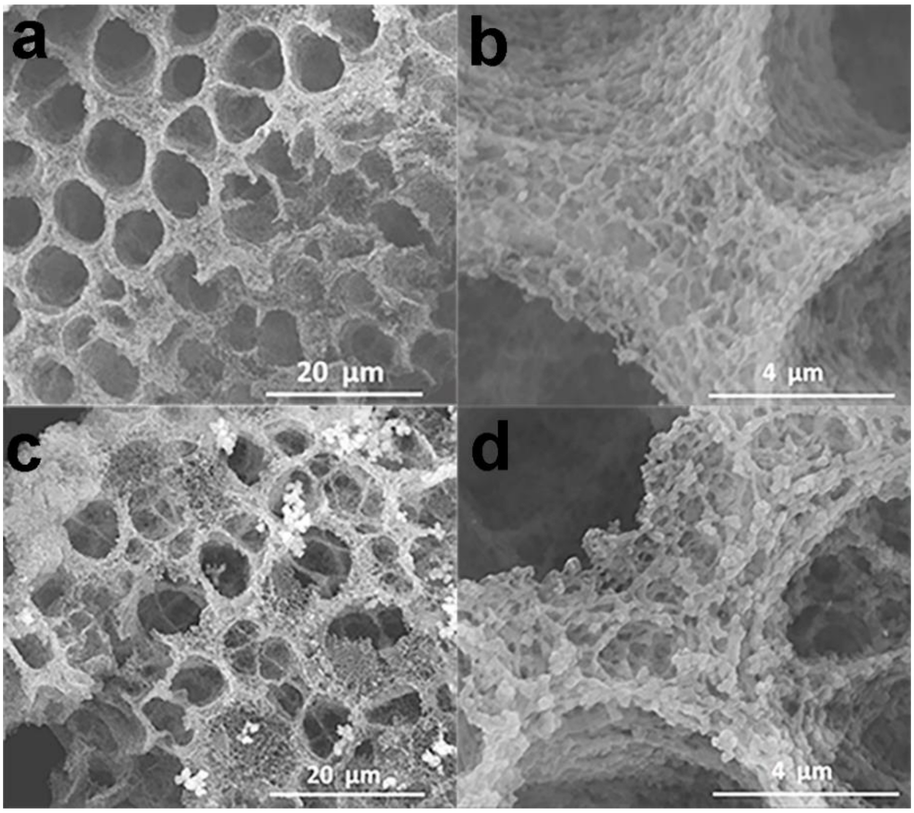

Figure S1. SEM images of a, b) Co@PAN-A and c, d) Co@PAN-B.

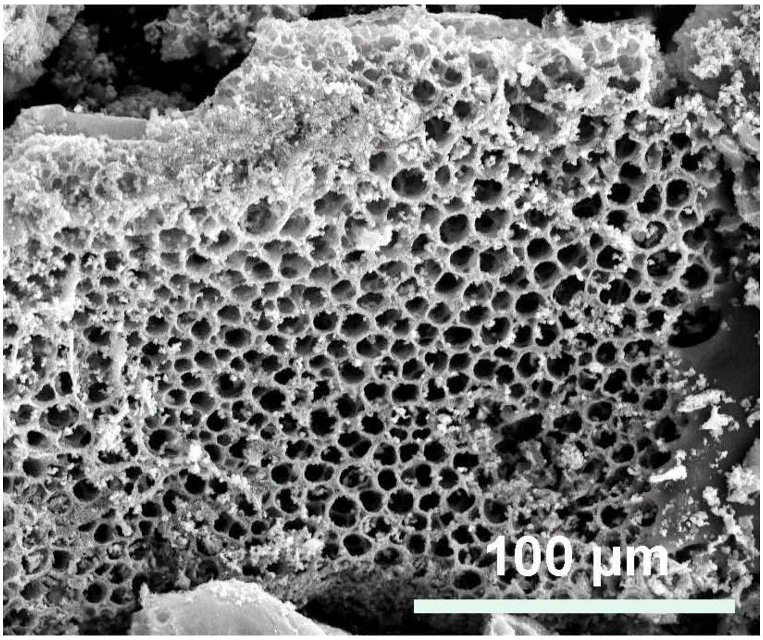

Figure S2. SEM image of CS@PC composite at low magnification.

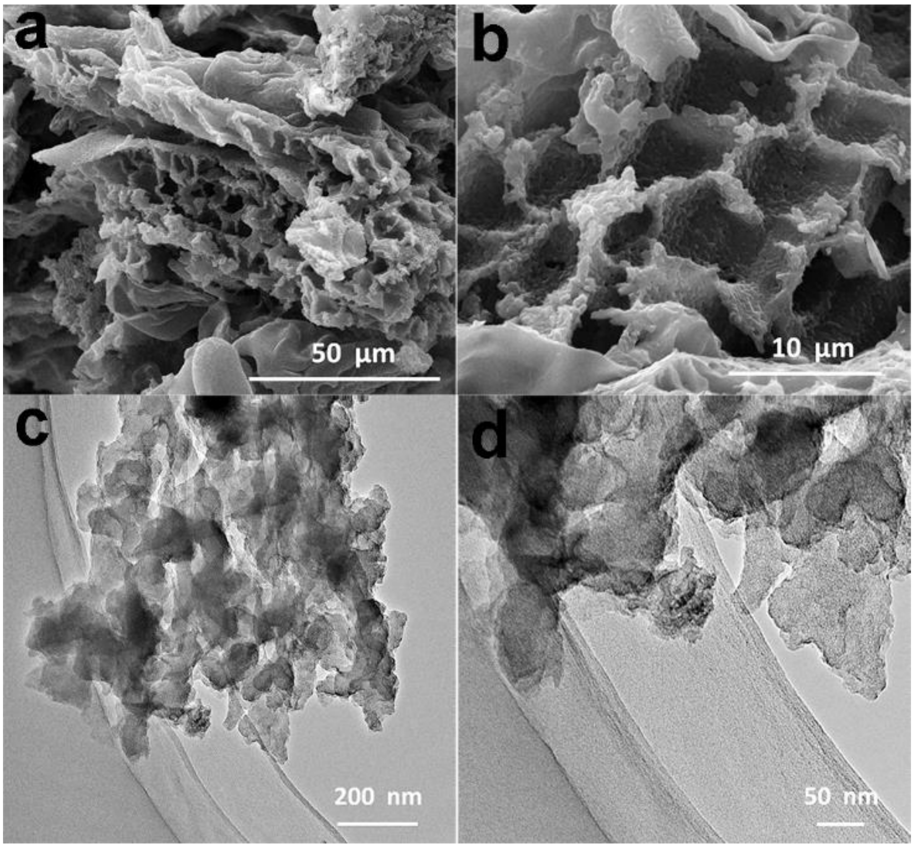

Figure S3. a, b) The SEM images and c, d) TEM images of PC.

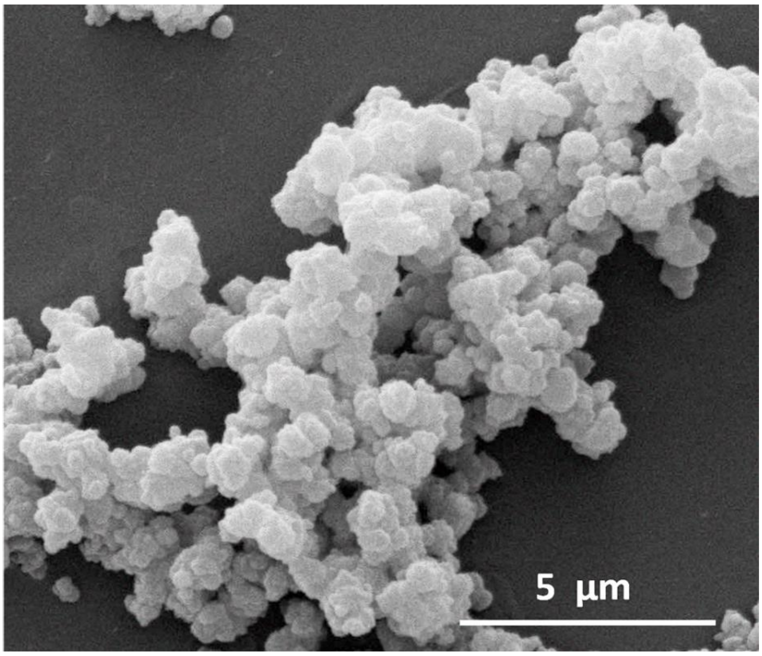

Figure S4. The SEM image of CS sample.

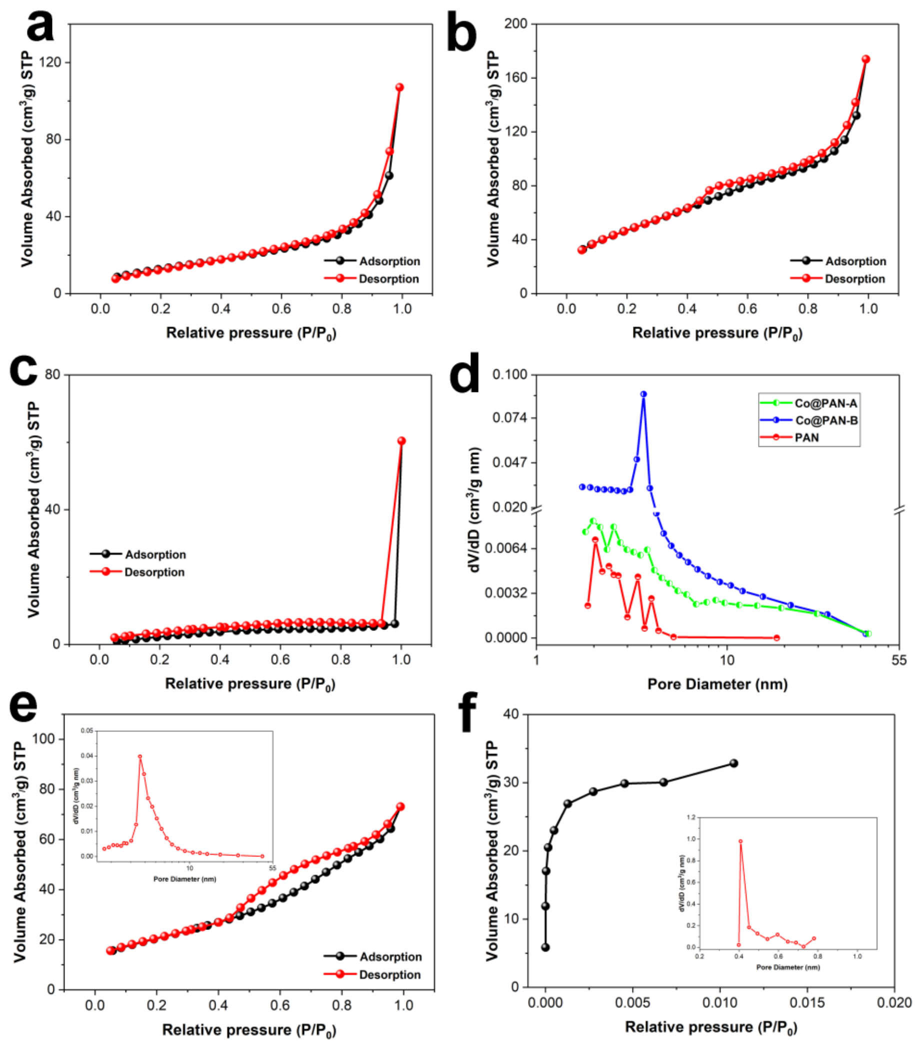

Figure S5. Nitrogen adsorption-desorption isotherms of a) Co@PAN-A, b)Co@PAN-B and c)PC. d) The corresponding pore-size distributin curves of the three samples. e) Nitrogen adsorption-desorption isotherm of CS sample and pore-size distribution (inset of e). f) The nitrogen adsorption isotherm of CS@PC at $P/P_0 < 0.05$ and micropore distribution (inset of f).

Table S1. Refined unit cell lattice parameters of CoS, Co_9S_8, and $Co_{1-x}S$ in CoS_x composite and standard data of CoS (ICSD Card No. 624857), Co_9S_8 (ICSD Card No. 660368), and $Co_{1-x}S$ (ICSD Card No. 42703).

Sample	Lattice parameters					Phase content (wt. %)	wRp (%)
	a (nm)	b (nm)	c (nm)	γ (°)	V (nm^3)		
CoS in CoS_x composite	0.33720	0.33720	0.51724	120.000	0.05093	75.850	7.76
CoS	0.33680	0.33680	0.51700	120.000	0.05079	—	—
Co_9S_8 in CoS_x composite	0.99242	0.99242	0.99242	90.0000	0.97743	20.164	7.76
Co_9S_8	0.99273	0.99273	0.99273	90.0000	0.97835	—	—
$Co_{1-x}S$ in CoS_x composite	0.35952	0.35952	0.58091	120.000	0.06503	3.986	7.76
$Co_{1-x}S$	0.36121	0.36121	0.57752	120.000	0.06525	—	—

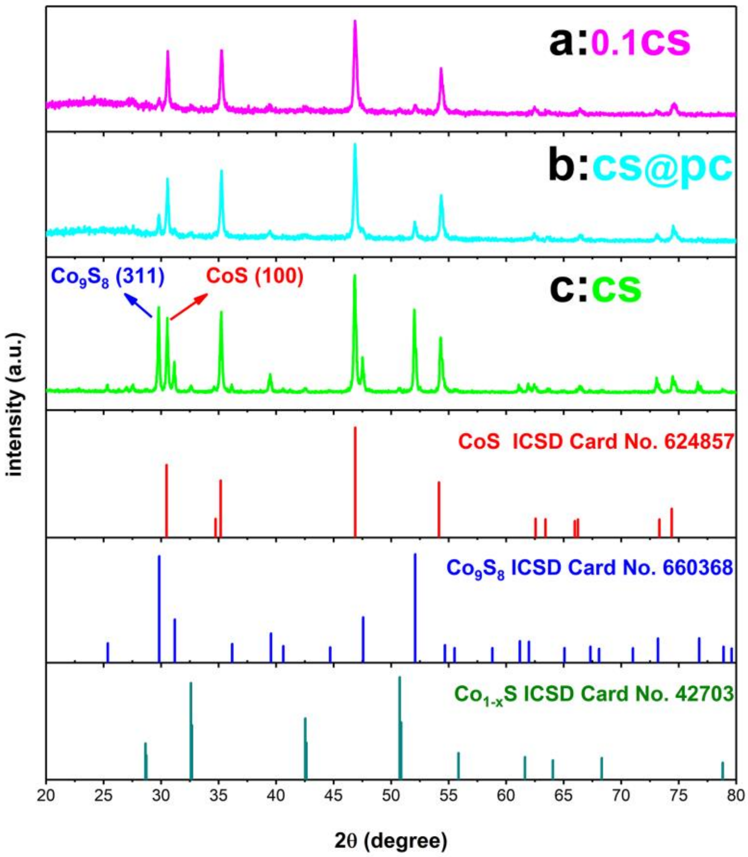

Figure S6. The XRD patterns of a) 0.1CS, b) CS@PC and c) CS.

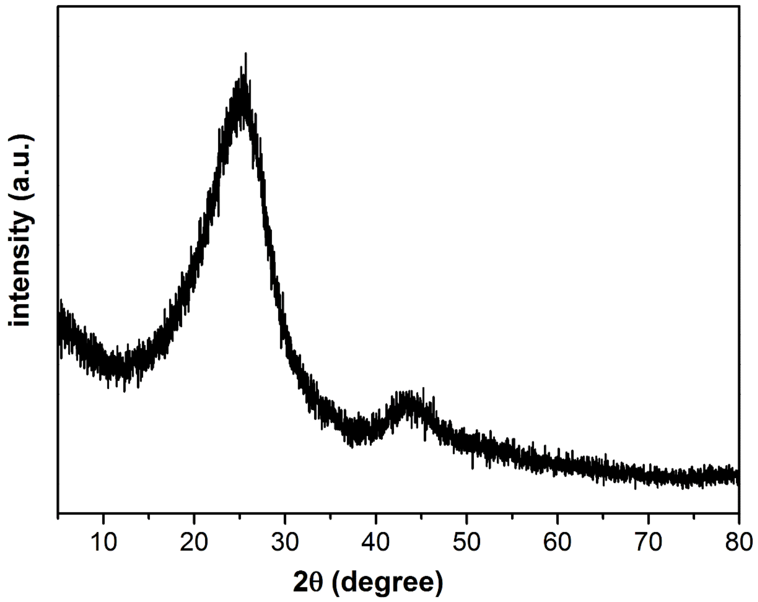

Figure S7. The XRD pattern of PC.

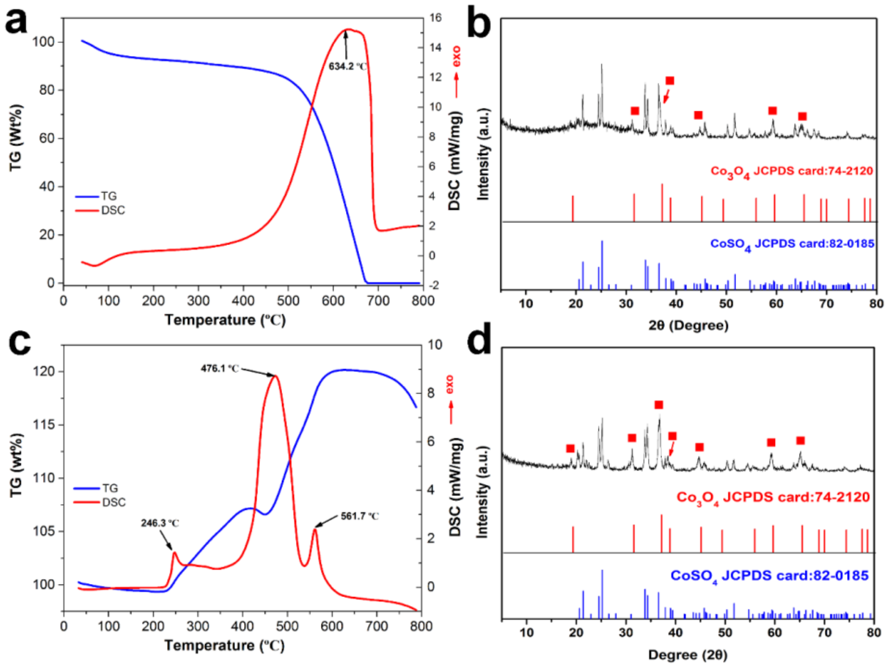

Figure S8. TG-DSC curves of a) PC and c) CS. XRD patterns of the residual material after TG-DSC tests of b) CS@PC and d) CS.

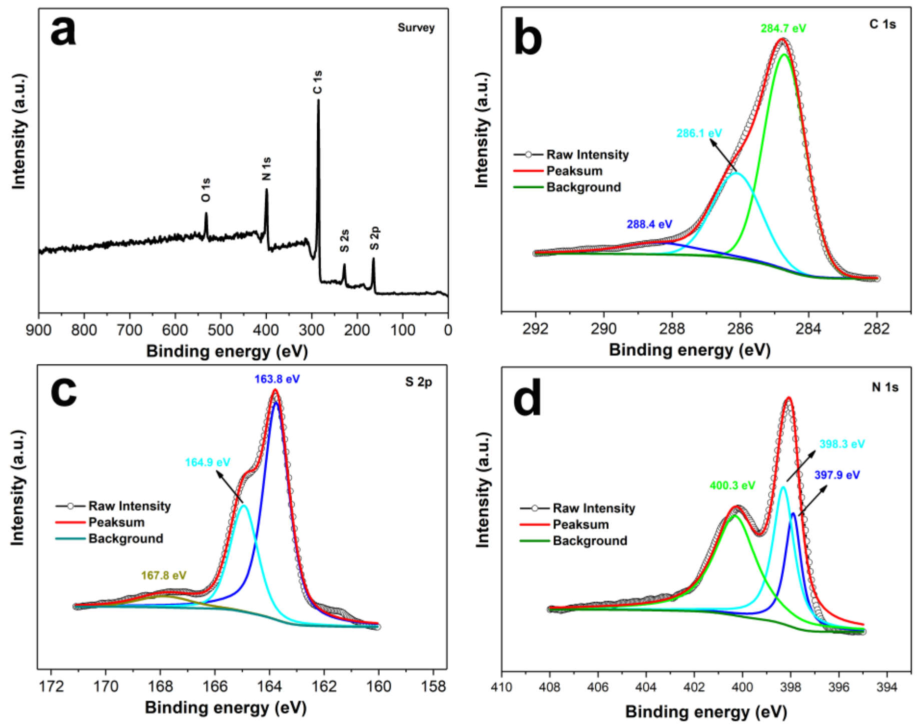

Figure S9. a) Typical XPS survey spectrum and the corresponding b) C 1s, c) S 2p, d) N 1s spectra of the pure carbon annealed with sulfur powder. The survey spectrum shows that the C, N, O, S element coexist in the carbon annealed with sulfur powder, except Co. The C 1s and N 1s spectra are similar to that of the CS@PC. And the high-resolution spectrum of S 2p has two main peaks at binding energy 163.8 eV and 164.9 eV, corresponding to C-S $2p_{3/2}$ and $2p_{1/2}$, respectively.

Table S2. Elemental compositions of CS@PC and PC-S.

Samples	C_{xps} (At %)	N_{xps} (At %)	S_{xps} (At %)	O_{xps} (At %)	Co_{xps} (At %)
CS@PC	60.03	10.12	10.84	14.37	4.65
PC-S	72.67	15.76	7.27	4.3	0

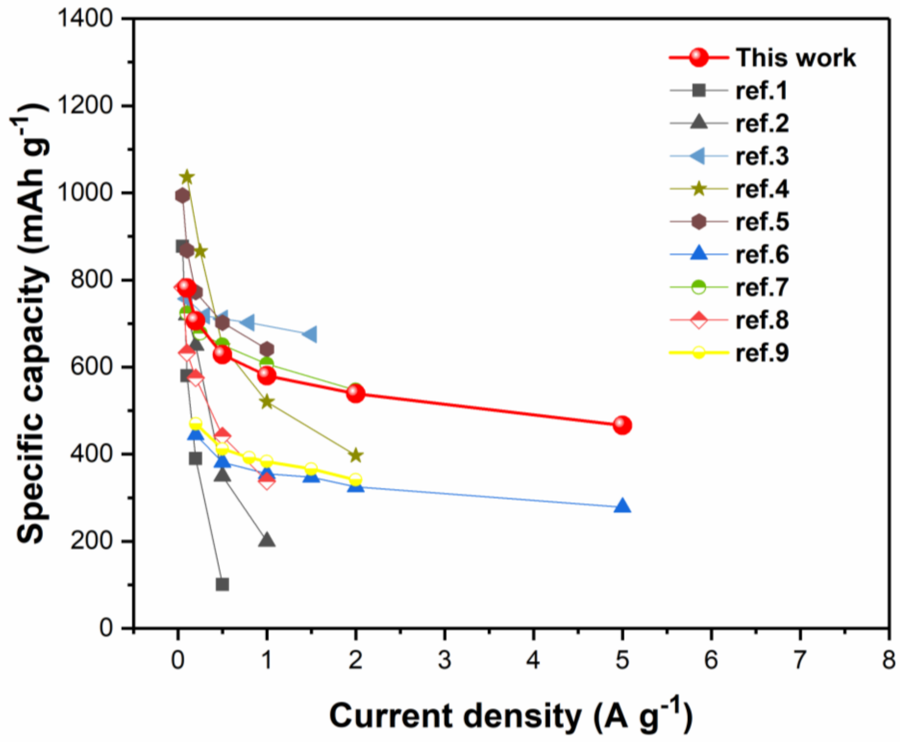

Figure S10. A survey of electrochemical properties of cobalt sulfide anodes in lithium ion batteries.

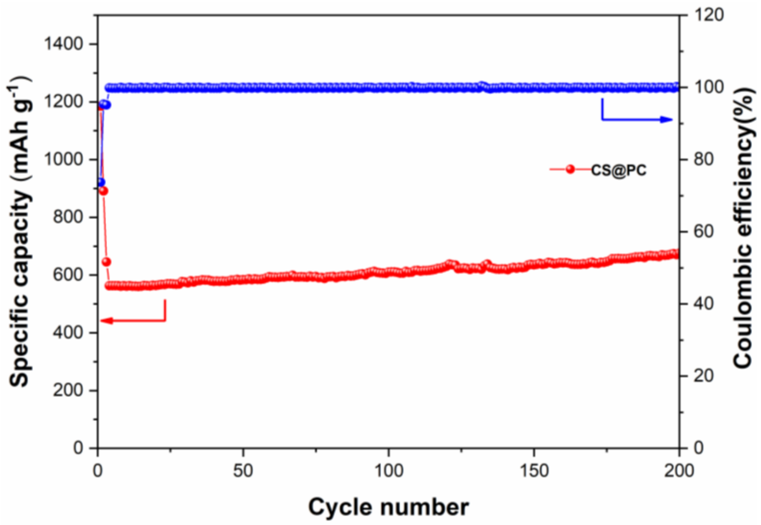

Figure S11. The cycling performance of the CS@PC electrode with 1.1 mg cm^{-2} mass loading of the active material at the current density of 1000 mA g^{-1} between 0.01 and 3 V.

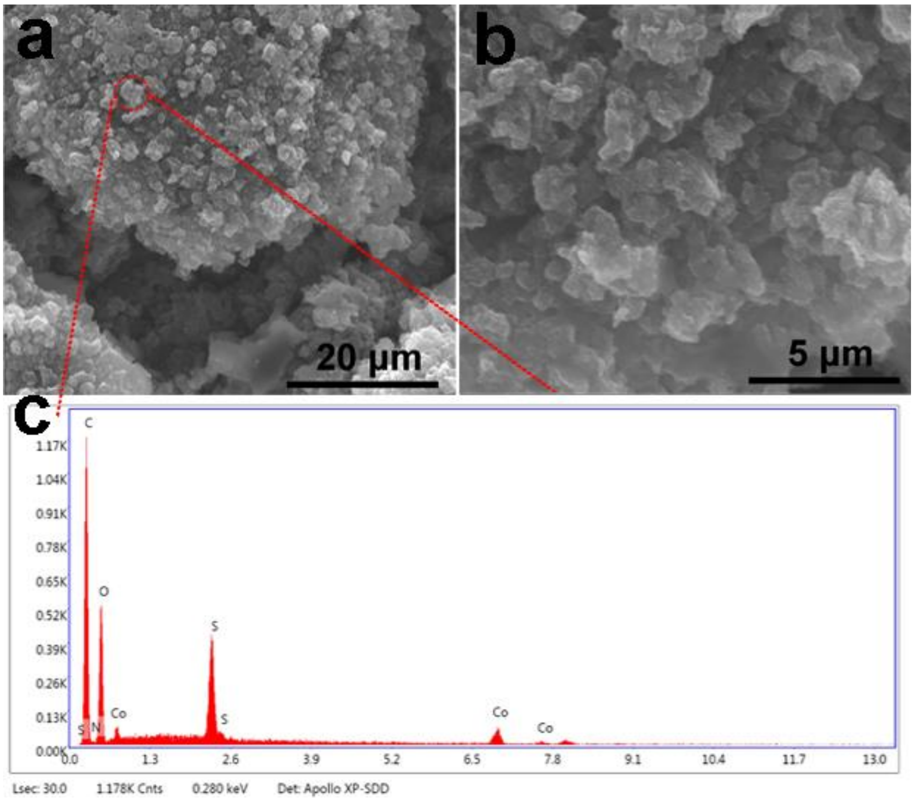

Figure S12. a, b) SEM images of CS@PC after 500 cycles at 1000 mA g^{-1} at different magnifications. c) The EDX spectrum of the selected region in the picture a (red dotted circle).

Table S3. The EIS fitting parameters of CS@PC, PC and CS samples.

Samples	R_s (Ω)	R_f (Ω)	R_{ct} (Ω)
CS@PC	4.779	10.86	138.1
PC	4.713	5.539	106.4
CS	4.775	13.5	276.7

The calculation formula is shown as follows:

$$D = \frac{R^2T^2}{2A^2n^4F^4C^2\sigma^2} \qquad \text{Equation S1}$$

Where R represents the gas constant, T is the test temperature, A is the surface area of electrode, F is the Farady constant, *n* represents the number of electrons per molecule attending the charge-discharge reaction, C is the concentration of lithium ion in our composite electrode, and σ is the slop of the line $Z' - \omega^{-1/2}$ (shown in Figure 7b).

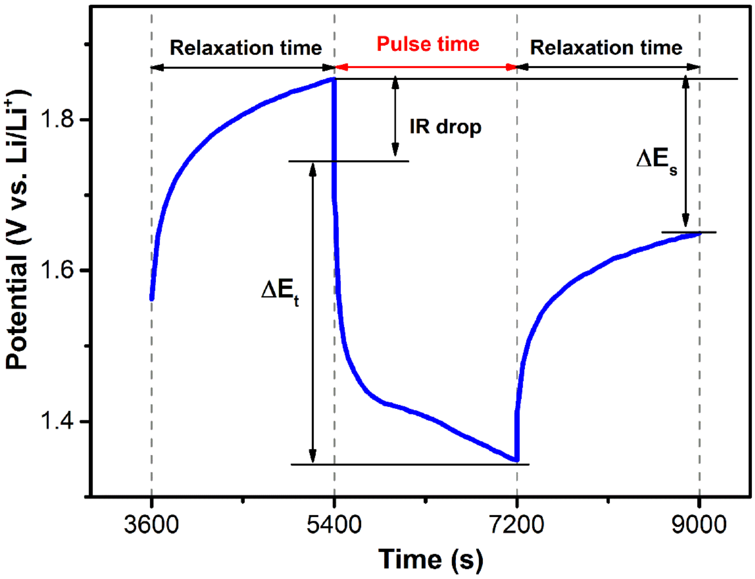

Figure S13. E vs. t curves of CS@PC electrode for a single GITT during discharge process.

The lithium diffusion coefficient was measured by using Galvanostatic intermittent titration technique (GITT) and calculated based on equation S2 as follows.

$$D = \frac{4L^2}{\pi\tau}\left(\frac{\Delta E_S}{\Delta E_t}\right)^2 \qquad \text{Equation S2}$$

Where t is the duration of the current pluse (s), τ is the relaxation time (s), and ΔE_s is the steady-state potential (V) by the current pluse. ΔE_t is the potential change (V) during the constant current pluse after eliminating the *i*R drop (Figure S13). L is lithium ion diffusion length (cm); for compact electrode, it is equal to thickness of electrode.

References

[1] R. Jin, J. Zhou, Y. Guan, H. Liu, G. Chen, J. Mater. Chem. A **2014**, *2*, 13241.

[2] Y. Wang, J. Wu, Y. Tang, X. Lü, C. Yang, M. Qin, F. Huang, X. Li, X. Zhang, ACS Appl. Mater. Interfaces **2012**, *4*, 4246.

[3] Y. Ko, S. Choi, S. Park, Y. Kang, Chem. -Asian J. **2014**, *9*, 572.

[4] X. Zhang, X. Liu, G. Wang, H. Wang, J. Colloid Interface Sci. **2017**, *505*, 23.

[5] J. Guo, F. Li, Y. Sun, X. Zhang, L. Tang, Electrochim. Acta **2015**, *167*, 32.

[6] F. Han, C. Zhang, B. Sun, W. Tang, J. Yang, X. Li, Carbon **2017**, *118*, 731.

[7] Z.P. Li, W. Li, H. Xue, W. Kang, X. Yang, M. Sun, Y. Tang, C. Lee, RSC Adv. **2014**, *4*, 37180.

[8] Z. Huang, Y. Wang, J. Wong, W. Shi, H. Yang, Electrochim. Acta **2015**,*167*, 388.

[9] F. Han, C. Tan, Z. Gao, J. Power Sources **2017**, *339*, 41.
